# Membranous Obstruction of the IVC: Role of Advanced Imaging and Intravascular Ultrasound

**DOI:** 10.1016/j.jaccas.2025.103785

**Published:** 2025-06-18

**Authors:** Rohan Naik, Muhammad Yasir Adeel, Khagendra Dahal, Bruce A. Rheaume, Juyong Lee

**Affiliations:** a Pat and Jim Calhoun Cardiovascular Center, School of Medicine, University of Connecticut, Farmington, Connecticut, USA; b Division of Cardiology, Creighton University, Omaha, Nebraska, USA

**Keywords:** endovascular intervention, IVC membrane, IVC obstruction, IVC web, IVUS

## Abstract

**Background:**

Inferior vena cava (IVC) obstruction is an underdiagnosed clinical entity. Recognizing obstruction of the IVC can be challenging due to its insidious and often subclinical presentation.

**Case Summary:**

A 66-year-old woman presented with chronic bilateral lower extremity swelling. An evaluation revealed septate membranes within the IVC that were causing IVC obstruction. This was successfully treated with membranotomy using balloon angioplasty.

**Review:**

We discuss our diagnostic approach and highlight the utility of intravascular ultrasound in making a clinical diagnosis and in guiding management.

## Visual Summary

**Figure:**
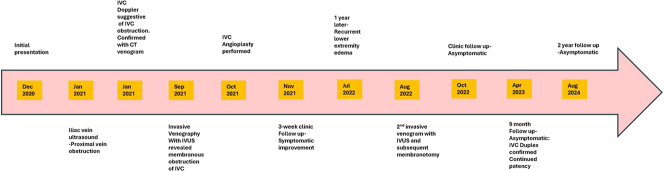

## Case Description

**Figure 1:**
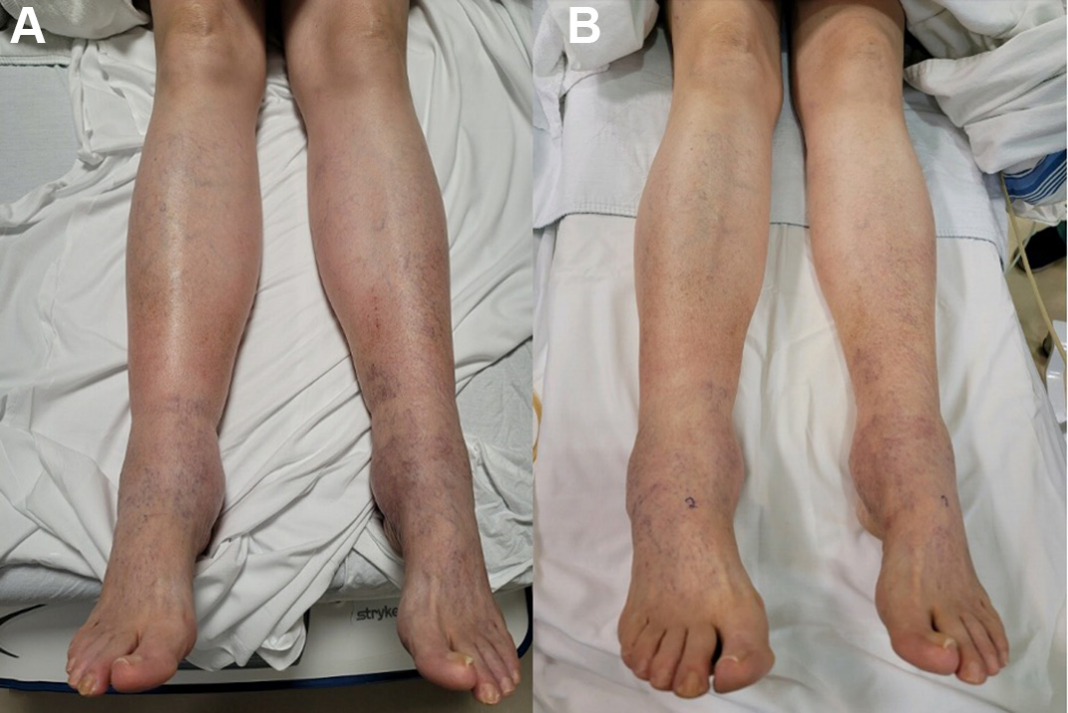
Physical Examination of the Lower Extremities Before and After Angioplasty (A) Inspection of lower extremities during initial presentation reveals bilateral leg edema extending from foot to lower thigh. Telangiectasias are visible over the foot and calf with hyperpigmentation and erythematous changes consistent with venous hypertension. (B) Inspection of lower extremities after balloon angioplasty reveals reduced swelling with less erythema and fewer visible dilated vessels.

A 66-year-old White woman presented to the vascular cardiology clinic with a chief complaint of bilateral erythematous lower extremity edema for about 1 year (Figure 1A). The edema in her legs worsened with prolonged standing and improved with exercise. She reported that her lower extremities felt very heavy, “almost like lead.” There was no history of recent trauma or immobility. She denied experiencing symptoms of shortness of breath, orthopnea, or paroxysmal nocturnal dyspnea. She had not had a recent fever or chills and did not report recent travel outside the United States.

**Take-Home Messages**

• IVC obstruction is an underdiagnosed clinical condition in which lower extremity swelling may be the only presenting symptom.
• Iliac vein and IVC duplex ultrasonography are useful initial diagnostic tests, but CT venography and invasive venography with IVUS can confirm the diagnosis.
• Membranotomy by balloon angioplasty, with or without metallic stenting, is an effective and durable treatment.

The physical examination revealed vital signs within normal limits. Her body mass index was 23.24 kg/m^2^. Inspection of her lower extremities revealed 3+ bilateral pitting edema of her legs, extending from her feet to her thighs. The swelling was slightly more prominent on the right side. Several telangiectasias were visible over her feet and calves with hyperpigmentation and erythematous changes consistent with venous hypertension. The cardiac and pulmonary examination was unremarkable. The abdominal examination showed no evidence of mass, ascites, or organomegaly.

Her past medical and surgical history included right-sided breast carcinoma, status postmastectomy; precancerous endometrial lesion, status posthysterectomy; and bilateral salpingo-oophorectomy, gastroesophageal reflux disease, vitamin B_12_ deficiency, and an inguinal hernia repair. She had no medical history of deep vein thrombosis (DVT), pulmonary embolism, or radiation therapy. Her medications included pantoprazole and cyanocobalamin. She had a family history of varicose veins and breast cancer. She was a lifelong nonsmoker and did not use any illicit substances.

Our initial differential diagnosis was broad and included local causes, such as chronic venous insufficiency or venous reflux disease; nonthrombotic iliac vein lesions, including both etiologies of intrinsic (membranes) and extrinsic compression (by overlying artery, as in May-Thurner syndrome); secondary lymphedema due to pelvic compression of vascular structures (postsurgical adhesions or malignancy); and postthrombotic syndrome due to sequelae of DVT. Additional considerations included diagnoses related to increased hydrostatic pressure, such as congestive heart failure (predominantly right-sided heart failure), early hepatic cirrhosis, and nephrotic syndrome. Pretibial myxedema and thyroid dysfunction also warranted exclusion.

Laboratory evaluations of complete blood counts, renal function, hepatic synthetic function, and thyroid function were found to be within normal limits. A transthoracic echocardiogram revealed preserved biventricular function without evidence of pulmonary hypertension or significant valvular heart disease. Duplex ultrasound of bilateral lower extremities excluded DVT but did demonstrate reflux within the right-sided short saphenous vein and deep veins. The patient may have had a component of phlebolymphedema due to chronic venous reflux disease, but the extremity edema seemed unusually severe to be explained on that basis alone. Therefore, further investigation was pursued to evaluate for potential nonthrombotic iliac vein lesions and to exclude postsurgical adhesions causing secondary lymphedema.

**Figure 2:**
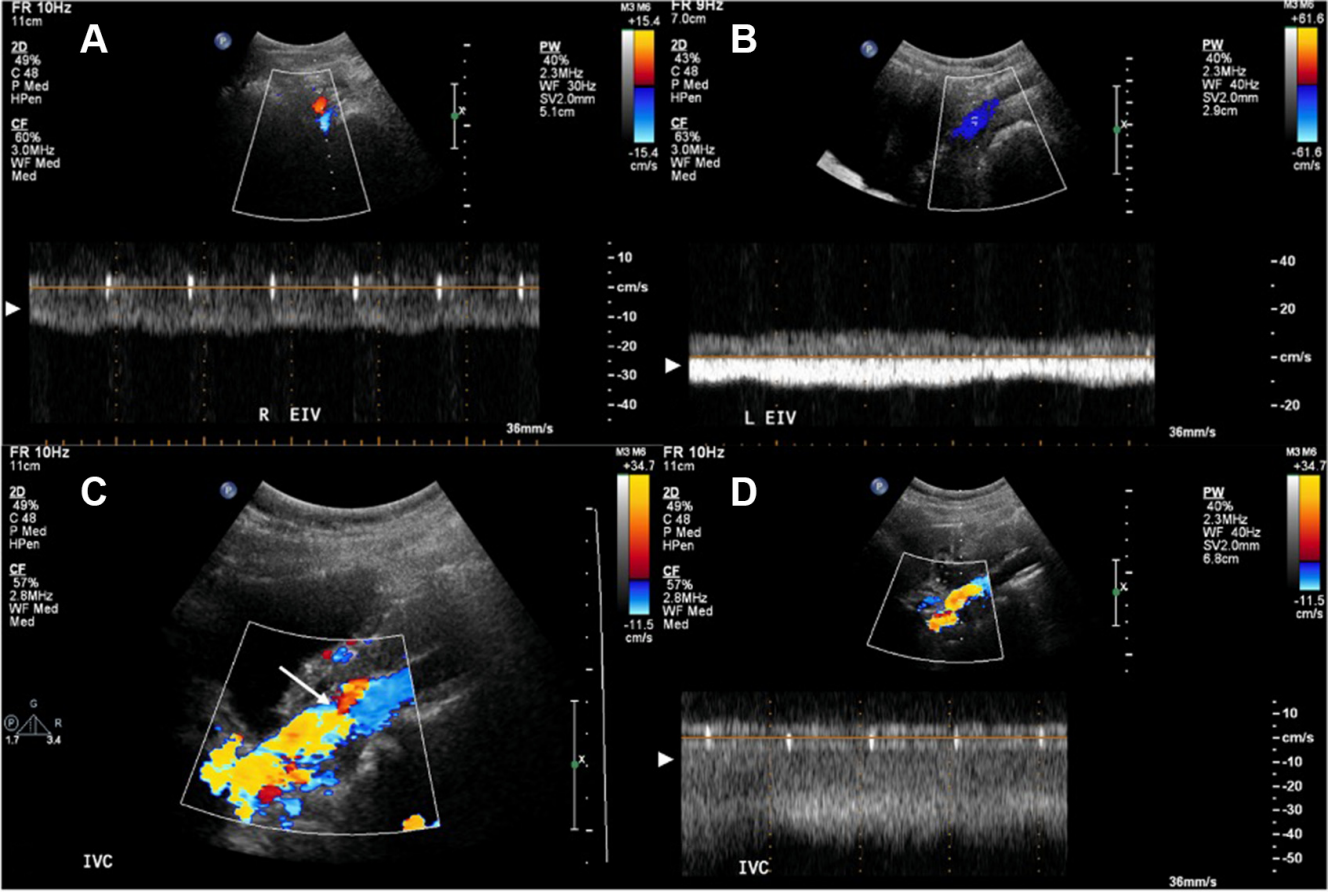
Bilateral External Iliac Vein and Inferior Vena Cava Doppler Ultrasound Doppler ultrasounds of the (A) right and (B) left external iliac veins shows continuous flow without respirophasicity, suggestive of a proximal occlusive lesion. Doppler ultrasounds of the inferior vena cava show high Doppler color-flow with (C) aliasing and an (D) abnormal continuous waveform pattern concerning for inferior vena cava obstruction.

Ultrasound of the iliac veins was performed to rule out compression stenosis. The Doppler study revealed continuous flow without respirophasicity in the left and right iliac veins, suggestive of a proximal occlusive lesion (Figures 2A and 2B). Doppler ultrasound of the mid-inferior vena cava (IVC) demonstrated high Doppler color-flow with aliasing (Figures 2C and 2D), suggestive of a flow restrictive condition such as IVC stenosis or obstruction.

**Figure 3:**
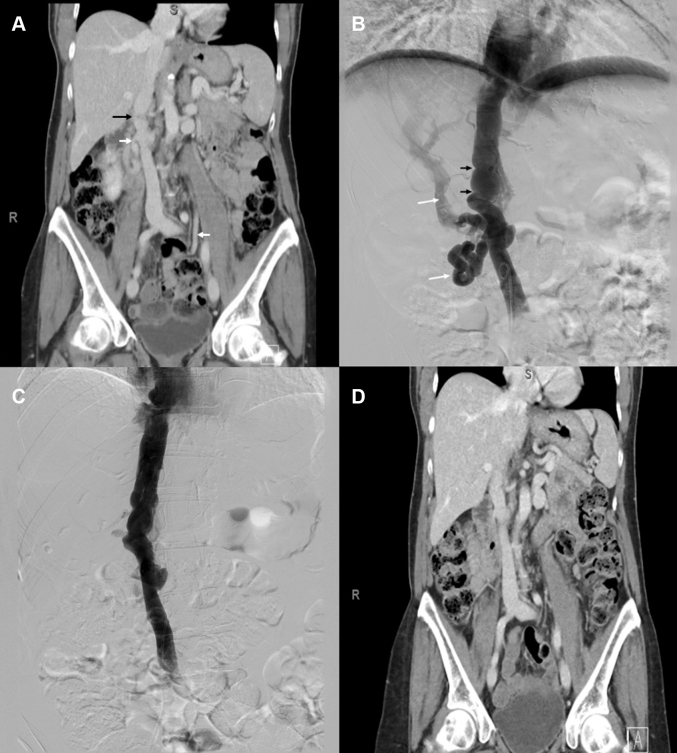
Computed Tomography Venography and Catheter Venography (A) Coronal view of the preprocedure computed tomography (CT) venogram of the abdomen and pelvis shows high-grade stenosis of the infrahepatic inferior vena cava (IVC) (black arrow) with numerous venous collaterals (white arrow). (B) Invasive venography before percutaneous intervention demonstrates multifocal IVC stenosis (black arrow) with severe collateral development (white arrows) in the infrahepatic IVC. (C) Venogram performed immediately after balloon angioplasty shows disappearance of venous collaterals with improved flow through the IVC. (D) Coronal view of CT venogram during follow-up shows continued resolution of collaterals.

The etiology of flow restriction within the IVC remained uncertain, so the patient underwent a computerized tomographic (CT) venogram of the abdomen and pelvis (Figure 3A). This study excluded a pelvic mass and adhesions but did reveal a high-grade stenosis of the infrahepatic IVC. Numerous venous collaterals had formed in the perineal, pelvic, and paralumbar region, extending into the upper abdomen. The stenosis of the IVC appeared to be the result of an intraluminal process.

**Figure 4:**
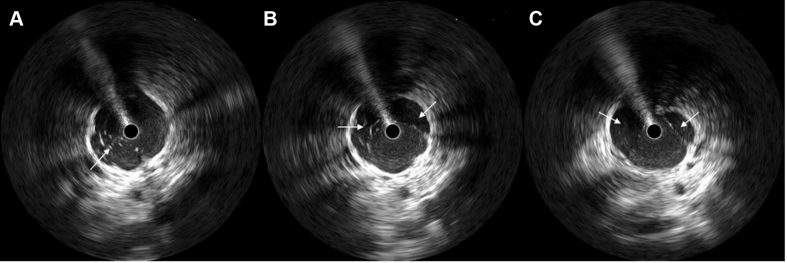
Intravascular Ultrasound of the Infrahepatic Inferior Vena Cava (A to C) Intravascular ultrasound (IVUS) imaging reveals multiple septate membranes (white arrows) within different segments of the infrahepatic inferior vena cava (the circle in the center of the vessel indicates the IVUS catheter).

The patient underwent invasive catheter venography with intravascular ultrasound (IVUS) imaging of the iliac veins using an IVUS OptiCross catheter (Boston Scientific). The inferior venacavogram demonstrated severe multifocal stenosis and severe collateral development in the infrahepatic IVC (Figure 3B). IVUS imaging performed from the right iliofemoral vein up to the right atrium revealed multiple septate membranes with severe stenosis in the region of the infrahepatic IVC (Figures 4A to 4C, Video 1).

IVC angioplasty was performed with a 16 × 60 mm XXL Balloon Dilation Catheter (Boston Scientific) twice in 2 locations, which resulted in complete dilatation of the proximal segment but partial correction of the distal luminal stenosis. There was marked improvement in flow through the IVC accompanied by a significant reduction in the size of venous collaterals and collateral flow (Figures 3C and 3D).

At the 3-week clinical follow-up evaluation, the patient reported significant improvement in her presenting symptoms. A year later, she developed recurrent edema, albeit to a lesser degree. The patient was re-evaluated with invasive venography and IVUS. Venography showed an area of possible narrowing in the IVC along with collaterals. IVUS revealed residual membranous obstructions below the site of the prior angioplasty, which was likely responsible for the flow obstruction and resultant symptoms.

**Figure 5:**
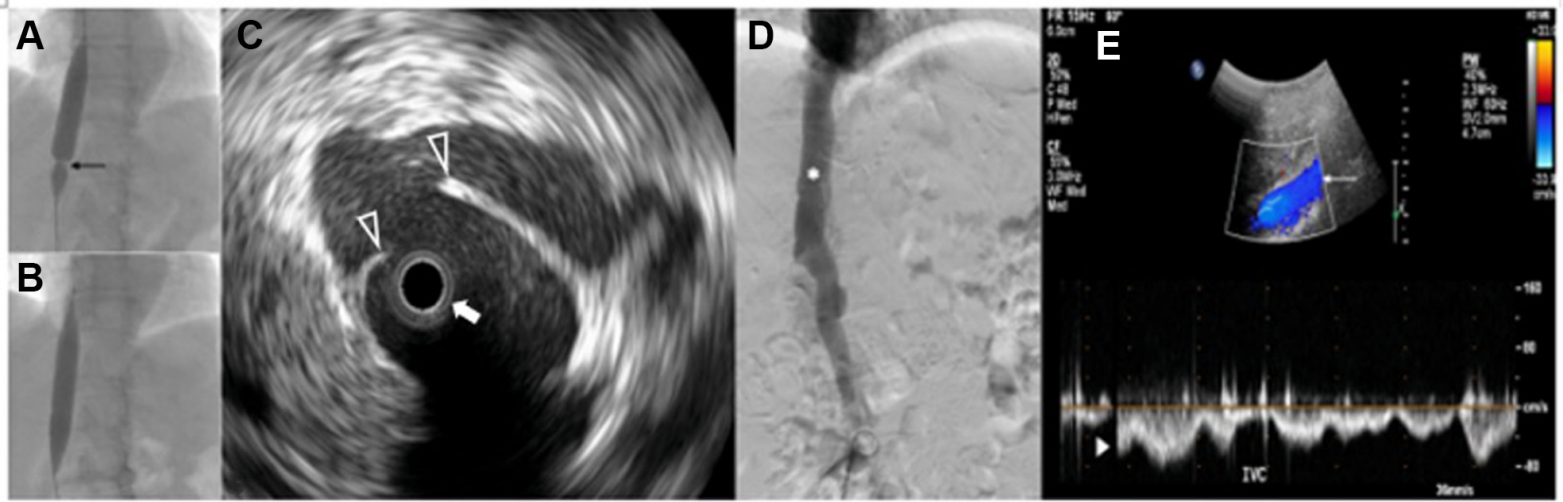
Second Membranotomy Procedure (A) Membranous obstruction (black arrow) is seen indenting the peripheral angioplasty balloon during inflation. (B) Balloon angioplasty with membranotomy successfully disrupted the IVC membrane, as is demonstrated by the fully expanded balloon. (C) A membranous tear (unfilled arrowheads) is identified by IVUS imaging (filled white arrow denotes the IVUS catheter). (D) Venographic imaging shows a normal forward flow within the inferior vena cava (IVC), with complete disappearance of collateral vessels (white star denotes IVC). (E) Duplex ultrasound of the IVC performed 9 months later exhibits patent IVC without areas of high flow or aliasing of color Doppler (white arrow), along with the return of a normal respirophasic Doppler pattern (white arrowhead). Abbreviations as in Figures 3 and 4.

The patient underwent balloon angioplasty with a 16 × 60 mm XXL Balloon Dilation Catheter (Boston Scientific) which acted as a membranotomy. The membranous obstructions that were not dilated in the prior procedure were completely dilated at this time (Figures 5A and 5B). This was subsequently confirmed with IVUS, which revealed ruptured membrane (Figure 5C). The venography showed the disappearance of membranous lines and normalized antegrade flow with resolution of collateral vessel flow (Figures 5C and 5D).

**Visual Summary:**
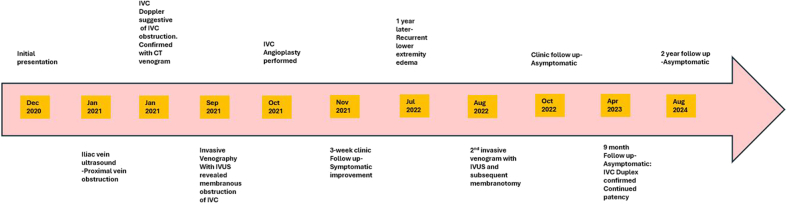
Treatment Timeline for Membranous Obstruction of the IVC

Duplex ultrasound of the IVC performed 9 months after the second angioplasty procedure demonstrated a normalized IVC Doppler pattern compared with the prior study, which was consistent with continued patency of the IVC (Figure 5E). Over the subsequent year and up to the present day, the patient reports significant reduction in lower extremity swelling, the ability to ambulate without physical limitation or symptoms, and an overall improvement in quality of life. On follow-up physical examination, her lower extremities appeared less erythematous and less edematous (Figure 1B).

## The Mini-Review

IVC obstructions are rare, but they are also underrecognized. The incidence appears to be higher in Asia compared with Western countries.^1^^,^^2^ The underlying etiology of IVC obstructions includes congenital IVC anomalies, postthrombotic obstruction (associated with high-risk DVT), and benign IVC obstructions (membranes, trabeculations, or spurs).

Congenitally abnormal IVCs occur in 0.5%-1% of the general population and are associated with an increased risk of thrombosis.^3^ Among those with a congenitally normal IVC, IVC obstructions most commonly develop due to underlying prothrombotic factors (eg, thrombophilia, malignancy, smoking, oral contraceptives, hormone replacement therapy, nephrotic syndrome) or from anatomical pathologies, such as an abdominal mass causing extrinsic compression of the IVC, adhesions from prior surgery, renal cell tumors, Budd-Chiari syndrome, May-Thurner syndrome, or the thrombotic occlusion of an IVC filter.^3^ Finally, benign IVC obstructions may be caused by IVC membranes, as was the case in our patient. These are usually idiopathic and can impair venous drainage and predispose individuals to venous thrombosis.

The preponderance of benign IVC obstructions occurring in Japan, India, and South Africa (particularly affecting its Black population) raise the proposition of a multifactorial etiology that may include geographic, ethnic, environmental, and nutritional influences.^4^ Based on autopsy studies, benign IVC obstructions vary considerably in location and appearance from case to case.^4^ They may be present anywhere between the terminal portion of the IVC to the IVC–right atrial junction, including the hepatic portion of the IVC.^4^ These obstructions may appear thick or thin, be incomplete or complete, or exist as focal or more extensive obstructions spanning over 5 cm in length.^4^ Histological descriptions are scant, but there have been narratives and illustrations describing elastic fibers forming these membranous obstructions that are contiguous with the wall of the IVC; these structures were notably devoid of inflammatory cells and scar tissue.^4^

Benign IVC obstructions can be classified as membranous (type I: focal obstructions where the obstructed segment of IVC is ≤1.0 cm) or segmental (type II: obstructed segment of IVC is >1.0 cm).^1^^,^^5^ The clinical presentation can be variable, depending on whether the IVC obstruction develops acutely or chronically and the level of obstruction.^3^ Intrahepatic IVC obstruction may present as liver dysfunction, whereas infrahepatic IVC obstructions present primarily as leg edema.^6^ Patients commonly report symptoms of lower extremity heaviness, extremity pain or cramping, and scrotal swelling.^3^

Chronic IVC obstruction is associated with the formation of large venous collaterals. Although rare, the paraspinal veins can enlarge, causing compressive symptoms of sciatica, lumbar radiculopathy, or even cauda equina syndrome.^3^ Extensive collaterals that may develop arise from embryonic caval elements.^7^

Due to the well-developed collaterals, cases of IVC obstruction may remain subclinical for many years.^3^ Physical examination findings in these cases include limb edema, dilated veins, erythematous changes, and skin ulceration.^8^ The presence of significant leg swelling (beyond ankle edema) is not a feature of isolated saphenous vein reflux, and the presence of associated iliac vein or IVC outflow obstruction should be suspected.^7^ Dilated venous collaterals are often present over the flanks and the back.^6^ Some may have abdominal ascites. The absence of hepatojugular reflux with the application of abdominal pressure rules out a cardiac cause of ascites.^6^

A diagnosis of IVC obstruction can be challenging due to its nonspecific clinical presentation. The absence of clinical guidelines to aid in the diagnosis and management of this condition and the lack of widespread awareness make this an underdiagnosed condition. If IVC obstruction is suspected, evaluation with modalities such as duplex ultrasonography, CT or magnetic resonance venography, or catheter venography with IVUS imaging may be performed. The venographic appearance may be normal in some cases of IVC obstruction and nonthrombotic iliac vein lesion.^7^ Therefore, among patients with symptomatic clinical, etiologic, anatomical, and pathophysiological (CEAP) classification of chronic venous disease clinical class III or greater who have unrevealing venography, IVUS imaging should be employed due to its superior diagnostic accuracy.^7^

In our case, based on the morphology on IVUS imaging, a diagnosis of IVC obstruction caused by IVC membranes was made. These are idiopathic in etiology and can be associated with a predisposition to venous congestion and thrombosis. Thrombosis is very often the cause for development of symptoms later in life. Although thrombus was not identified on IVUS imaging, its presence during a previous time in the disease process cannot be excluded. It remains unclear whether thrombosis played a role. The CT venogram findings of vast venous collaterals are consistent with the chronic nature of IVC membranes.

The current literature supports endovascular treatment of such IVC membranes.^1^, ^2^, ^3^^,^^5^, ^6^, ^7^, ^8^, ^9^, ^10^, ^11^ Percutaneous transluminal balloon angioplasty is performed with or without metallic stenting. A review of the current data seems to support treating membranous (type I) obstructions with percutaneous angioplasty, with or without a metallic stent.^1^^,^^5^^,^^8^ Patency rates are good with angioplasty alone and do not seem to differ significantly based on whether stenting is performed.^8^^,^^9^ Metallic stenting is associated with a high incidence of neointimal hyperplasia. For segmental obstructions (type II), percutaneous transluminal balloon angioplasty with metallic stenting may provide better long-term results with improved stent patency.^1^^,^^5^

In our patient, we decided to pursue membranotomy by balloon angioplasty without stenting for 2 reasons. First, the obstruction was due to a venous membrane that was amenable to membranotomy, as was demonstrated by serial IVUS images depicting a torn, thick membrane with disappearance of smaller membranes. Second, the membranous obstruction was near the renal veins, which raised concern for potential compromise of renal vein flow with stenting.

Membranotomy by balloon angioplasty is an effective treatment strategy when there is certain membranous stenosis, as was seen with IVUS in our case. This was confirmed by the direct visualization of disrupted membranes within the IVC and the observation of improved vessel patency on IVUS (Figure 5C). Venography showed a dramatic disappearance of venous collaterals with improved forward flow through the IVC to the right atrium (Figure 5D). Chronic anticoagulation was prescribed due to an increased risk of DVT involving the distal venous tree among these patients.^7^

## Conclusions

IVC obstructions, especially membranous stenosis, require a high index of suspicion to diagnose. Iliac vein and IVC duplex ultrasound is an important initial diagnostic modality that can provide valuable diagnostic clues. Further evaluation with specialized tests, such as CT venogram and invasive venography with IVUS, can confirm the diagnosis. Percutaneous transluminal angioplasty with or without stenting is an effective and durable treatment modality.

## Funding Support and Author Disclosures

The authors have reported that they have no relationships relevant to the contents of this paper to disclose.

## Appendix

Video 1 Intravascular ultrasound shows multiple septate membranes with severe stenosis in the region of the infrahepatic inferior vena cava.
